# Supplementary material for: Saliva SARS-CoV-2 Antibody Prevalence in Children
Source: Microbiol Spectr. 2021 Sep 15;9(2):e00731-21. doi: 10.1128/Spectrum.00731-21 (PMC8557814; doi:10.1128/Spectrum.00731-21)

Supplementary Material Legends

Supplementary table 1. Mean, standard deviation, assay cut-off, replicability and precision of Luminex assays

Mean, standard deviation (SD) and assay cut-off (mean + 2x SD) are calculated and presented as $\log_{10}$ values. R^2 presents the data replicability and is calculated by simple linear regression on the data in this study plotted against a replicate measurement of data using the same assay conditions on a different day, using positive control sera (n=6) or saliva (n = 5) included on each plate. CV = coefficient of variation, the CV was calculated for each positive control sample and the range is shown.

Supplementary table 2. Study population characteristics and seroprevalence

Characteristics are described for all children included in at least one of the assays (n = 517). Prevalence was calculated as number (percentage) from non-missing values for either the Wantai RBD total antibody assay (n=487, 30 missing) or the independent variable. Abbreviations: COVID-19 = coronavirus disease 2019, ER = emergency room, No = number, PCR = polymerase chain reaction assay, RBD = Receptor binding domain, SARS-CoV-2 = severe acute respiratory syndrome coronavirus 2.

Supplementary figure 1. Participant recruitment and inclusion

The inclusion process is depicted for the total study sample (n=517). Differences in number of serum samples analyzed between the Luminex and the Wantai RBD total antibody assays are due to the serum volumes required for the Luminex (2,5 µl per sample) and the Wantai (200 µl per sample). Differences between total study sample and number of saliva samples are due to insufficient sampling.

Supplementary figure 2. IgG and IgA Titers in the Luminex assay

SARS-CoV-2 S, RBD and N protein specific IgG and IgA were measured in all serum (n=509) and saliva (n=430) samples with the Luminex assay, expressed in MFI. The cut-off for a positive result was determined using the geometric mean + 2x the geometric standard deviation, for each

combination of sample type, antibody isotype and antigen separately (see supplementary table 1). All dots represent individual data points, the black line is the geometric mean and the dotted line the cut-off. Abbreviations: S = trimeric SARS-CoV-2 spike protein, RBD = the monomeric receptor binding domain of the SARS-CoV-2 spike protein, N = SARS-CoV-2 nucleocapsid protein, MFI = median fluorescence intensity.

Supplementary figure 3. Serum and saliva antibody prevalence over time

SARS-CoV-2 S, RBD and N-specific IgG and IgA were measured in serum (n=487) and saliva (n=413) samples with the Luminex assay, expressed as MFI. Only samples also measured in the Wantai assay are shown with the positive Wantai results indicated in blue. The cut-off for a positive result in the Luminex was determined using the geometric mean + 2x the geometric standard deviation, for each combination of sample type, antibody isotype and antigen separately (see supplementary table 1). All dots represent individual data points, and the red dotted line is the assay cut-off. Data is plotted against the days since first inclusion. Abbreviations: S = trimeric SARS-CoV-2 spike protein, RBD = the monomeric receptor binding domain of the SARS-CoV-2 spike, N = SARS-CoV-2 nucleocapsid protein, MFI = median fluorescence intensity.

Supplementary tables

Supplementary table 1. Mean, standard deviation, assay cut-off, replicability and precision of Luminex assays

	Serum IgG			Serum IgA			Saliva IgG			Saliva IgA		
	**S**	**RBD**	**N**	**S**	**RBD**	**N**	**S**	**RBD**	**N**	**S**	**RBD**	**N**
Mean	0.30	0.59	0.99	0.54	1.52	0.93	0.90	0.82	0.75	2.40	2.60	2.75
SD	0.49	0.58	0.59	0.59	0.71	0.81	0.62	0.61	0.58	0.71	0.97	1.03
Cut-off	1.27	1.74	2.16	1.71	2.93	2.54	2.15	2.04	1.91	3.81	4.54	4.80
R^2	0.89	0.89	0.90	0.94	0.79	0.67	0.68	0.69	0.73	0.94	0.83	0.91
CV	1-4%	1-4%	1-4%	2-5%	2-5%	1-5%	2-8%	4-7%	4-9%	2-3%	2-5%	3-5%

Supplementary table 2 Study population characteristics and seroprevalence

	**Total sample (percentage)**	**Seronegative Wantai No (percentage)**	**Seropositive Wantai No (percentage)**
**Total sample**	517 (100%)	471 (96.7%)	16 (3.3%)
**Sex**			
Female	265 (51.3%)	242 (96.0%)	10 (4.0%)
Male	252 (48.7%)	229 (97.4%)	6 (2.6%)
**Age (years)**			
< 1	47 (9.1%)	34 (100.0%)	0 (0.0%)
1-4	71 (13.7%)	63 (98.4%)	1 (1.6%)
5-9	107 (20.7%)	99 (97.1%)	3 (2.9%)
10-14	146 (28.2%)	139 (95.9%)	6 (4.1%)
15-17	146 (28.2%)	136 (95.8%)	6 (4.2%)
**Inclusion**			
April/May	86 (16.6%)	75 (94.9%)	4 (5.1%)
May/June	109 (21.1%)	100 (97.1%)	3 (2.9%)
June/July	69 (13.3%)	64 (95.5%	3 (4.5%)
July/August	95 (18.4%)	88 (97.8%)	2 (2.2%)
August/September	107 (20.7%)	97 (96.0%)	4 (4.0%)
September/October	51 (9.9%)	47 (100.0%)	0 (0.0%)
**Immunocompromised state**	185 (35.8%)	176 (96.7%)	6 (3.3%)
- Immunodeficiency	2.9%		
- Autoimmune disease	30.8%		
- Hematological malignancies	0.8%		
- Use of immunomodulating drugs	34.7%		
**Underlying illness**	128 (24.8%)	114 (95.0%)	6 (5.0%)
- Obesity	2.9%		
- Respiratory	4.8%		
- Cardiovascular	6.2%		
- Diabetic	0.4%		
- Other malignancies	1.9%		
- Endocrine/metabolic	5.4%		
- Kidney disease	2.3%		
- Hematologic	2.1%		
- Psychomotor retardation	0.8%		
**No relevant medical history**	201 (38.9%)	180 (97.8%)	4 (2.2%)
Unknown	3 (0.6%)	3 (100.0%)	0 (0.0%)
**Type of hospital visit**			
- Day-care	271 (52.4%)	244 (94.9%)	13 (5.1%)
- Outpatient	103 (19.9%)	100 (99.0%)	1 (1.0%)
- ER visit	27 (5.2%)	27 (100.0%)	0 (0%)
- Inpatient	110 (21.3%)	94 (97.9%)	2 (2.1%)
- Unknown	6 (1.2%)	36 (100.0%)	0 (0%)

**Type of hospital**			
- Tertiary care center	363 (70.2%)	327 (95.3%)	16 (4.7%)
- Secondary care center	154 (29.8%)	144 (100%)	0 (0%)
**COVID-19 symptoms at time of inclusion**			
- Yes	138 (26.7%)	130 (98.5%)	2 (1.5%)
- No	377 (73.0%)	339 (96.0%)	14 (4.0%)
**COVID-19 symptoms in last 4 weeks**			
- Yes	232 (44.9%)	210 (94.6%)	12 (5.4%)
- No	280 (54.2%)	257 (98.5%)	4 (1.5%)
**COVID-19 in household**			
- Yes	10 (1.9%)	5 (50.0%)	5 (50.0%)
- No	497 (96.1%)	456 (97.6%)	11 (2.4%)
**Previous SARS-CoV-2 PCR Positive**	3/107 (2.8%)	1 (33.3%)	2 (66.7%)

Figures

Supplementary figure 1. Participant recruitment and inclusion

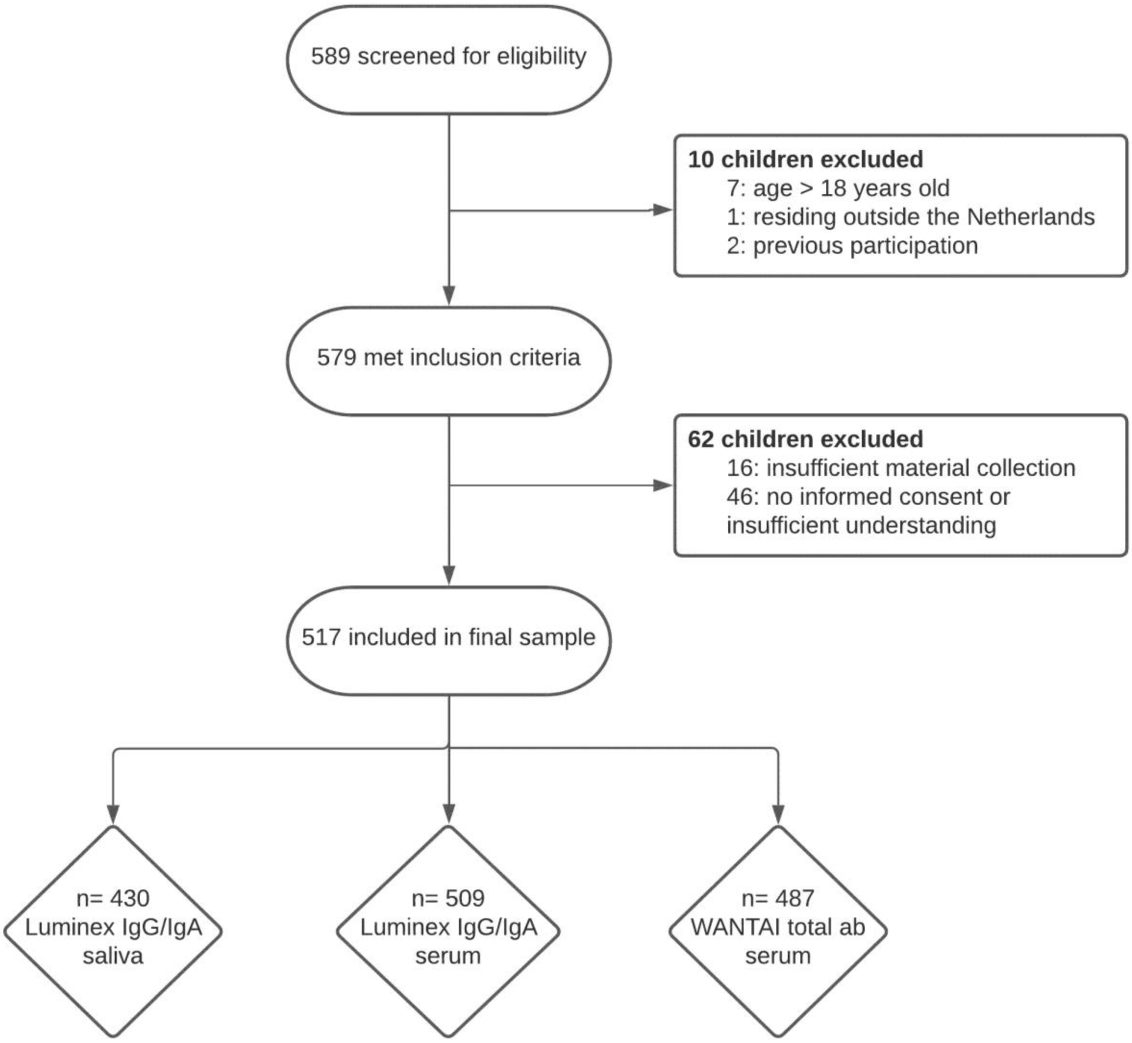

Supplementary figure 2. IgG and IgA Titers in the Luminex assay

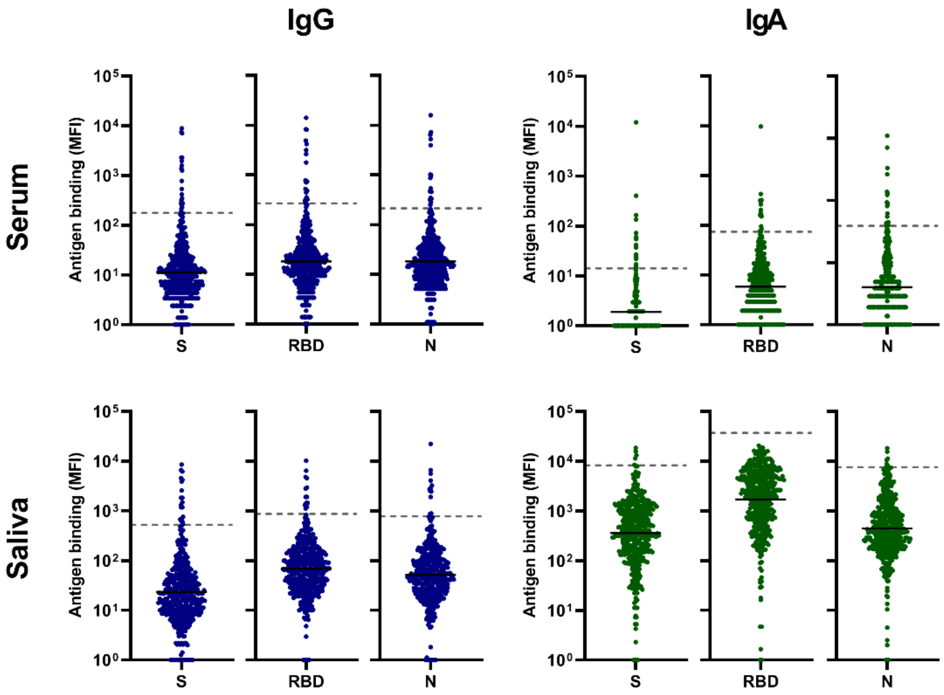

Supplementary figure 3. Serum and saliva antibody prevalence over time

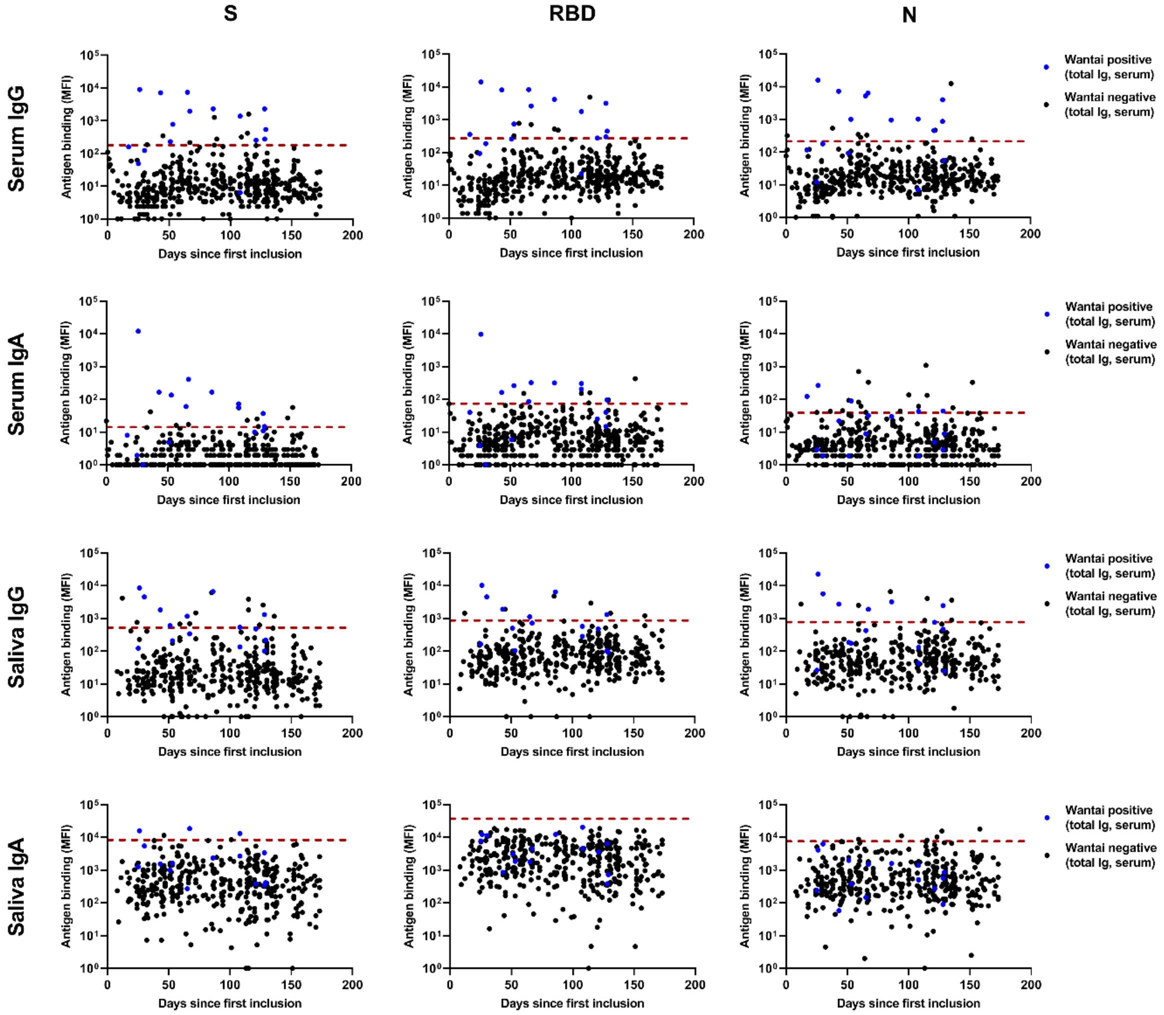

Supplement: SUPPLEMENTAL FILE 1 — Supplemental material. Download SPECTRUM00731-21_Supp_1_seq9.pdf, PDF file, 0.6 MB [file spectrum00731-21_supp_1_seq9.pdf]
